# Streetlight Control System Based on Wireless Communication over DALI Protocol

**DOI:** 10.3390/s16050597

**Published:** 2016-04-27

**Authors:** Francisco José Bellido-Outeiriño, Francisco Javier Quiles-Latorre, Carlos Diego Moreno-Moreno, José María Flores-Arias, Isabel Moreno-García, Manuel Ortiz-López

**Affiliations:** Department of Computer Architecture, Electronics and Electronic Technology, University of Córdoba, Córdoba 14071, Spain; el1qulaf@uco.es (F.J.Q.-L.); el1momoc@uco.es (C.D.M.-M.); jmflores@uco.es (J.M.F.-A.); p92mogai@uco.es (I.M.-G.); el1orlom@uco.es (M.O.-L.)

**Keywords:** street lighting system, wireless sensor networks, IEEE 802.15.4, DALI protocol

## Abstract

Public lighting represents a large part of the energy consumption of towns and cities. Efficient management of public lighting can entail significant energy savings. This work presents a smart system for managing public lighting networks based on wireless communication and the DALI protocol. Wireless communication entails significant economic savings, as there is no need to install new wiring and visual impacts and damage to the facades of historical buildings in city centers are avoided. The DALI protocol uses bidirectional communication with the ballast, which allows its status to be controlled and monitored at all times. The novelty of this work is that it tackles all aspects related to the management of public lighting: a standard protocol, DALI, was selected to control the ballast, a wireless node based on the IEEE 802.15.4 standard with a DALI interface was designed, a network layer that considers the topology of the lighting network has been developed, and lastly, some user-friendly applications for the control and maintenance of the system by the technical crews of the different towns and cities have been developed.

## 1. Introduction

Public lighting installations are important sources of energy consumption that are affected by certain factors, such as regulation and maintenance. This energy consumption can be reduced considerably by applying new communication and control technologies. The International Energy Agency (IEA) has estimated that the potential energy savings due to lighting technology can be approximately 133 to 212 TWh/y on a global level. The corresponding reductions in CO_2_ emissions could equal about 86–137 MT/y for IEA countries [1]. The European Commission carried out a recent study [2] proving that between 30% and 50% of the electricity used for lighting could be saved through research into energy-efficient lighting systems. The use of low-consumption lamps, lighting fixtures and other types of outdoor luminary lighting fixtures is advisable, as is the use of cleaner methods of generating electric energy. This is not just a profitable investment but can also improve the quality of the lighting [3].

Two-thirds of the public lighting systems currently in use are still based on obsolete and inefficient technologies, with higher energy consumption than needed; therefore, there is great technological potential to renovate public lighting and reduce energy consumption [4]. According to estimates, around 5% of the energy used in lighting is consumed by public lighting, which is the most important component of a city or town’s energy consumption.

The minimum acceptable or legal requirements for controlling lighting are different for industry, offices, streets, and public lighting, but the concept is always the same: a fixed lighting intensity must be always achieved with energy efficiency and the lowest possible cost for energy consumption to increase the expected lifespan of the lamps. As a result, by using available lamps, ballasts and control systems, as well as techniques to develop an efficient lighting system, it is possible to produce quality energy in a more efficient and profitable way [5].

Traditional energy-saving techniques entail totally or partially turning off streetlights, but this implies a reduction in the uniformity of the lighting, which also has a considerable impact on the lifespan of lamps.

Several recommendations exist to reduce the electric energy consumption of public lighting [6]. One of these actions is to control the on-off timespan of the lighting. This period can be controlled using photocell relays or astronomical time relays [7]. The photocells are activated or deactivated depending on the level of environmental light. Their main disadvantage is their low sensitivity to dawn and the presence of clouds, which can cause the relay to be activated too soon. On the other hand, astronomical time relays use a program that contains the sunrise and sunset times in each time zone. A second action is to reduce light output (dimming) during hours with reduced traffic by reducing the voltage applied to each lamp, which reduces electric energy consumption. Lastly, another proposed action is to use high-performance lamps and lighting devices. This consists of installing smart, adjustable electronic ballasts that are able to automatically detect electric failures and lamp problems; measure and send data, such as on the state of the lighting, the lighting level, the energy consumption, voltage, current, and power factor; and receive interruption and lighting control commands.

Regarding the need for uniform lighting, the control systems have two possibilities: light posts with two lamps or with only one fully controllable lamp. In the first case, one of the two lamps can be turned off late at night and during the early morning hours. In the second case, the lamp needs to be connected to a regulation system. The last method has two practical options: dimmable ballasts or centralized control systems as line voltage regulators.

On the other hand, remote management systems also exist that allow the user to control and monitor each lamp individually. When using these systems, the operator can monitor the main parameters of any light from a control room or a mobile unit. The obtained data can be processed, allowing for statistical calculation of the consumption, state of the lamp, voltage, failures, *etc*. In this way, the average repair time can be reduced. Another interesting parameter could be the arc voltage level, which entails the change from corrective maintenance to predictive maintenance, meaning economic savings in maintenance costs.

To optimize the control, the over-the-air management system needs to support bidirectional communication between the user and the ballast. This management system is implemented using a communication system and a control protocol for the lighting. The communication system can be wired or wireless. The wired communication systems that are normally used include Ethernet-based systems and those based on optical fiber or power-line carrier (PLC). As wireless standards, we can find, among others, GSM/GPRS, RF, Wi-Fi™, IEEE 802.15.4™ and ZigBee™. The latter two are responsible for the increase of wireless sensor networks (WSNs) [8,9].

Standard IEEE 802.15.4 is aimed at low-rate wireless personal area networks (WPANs) [10]. Its objective is to standardize the two lower layers of the communication protocols between open protocols, *i.e.*, the physical layer and the MAC layer. On the other hand, ZigBee defines the upper layers, the network and the application layers. For the lighting-control protocol, it is possible to choose between open protocols, such as TCP/IP, BACNet™, DMX 512™, LONWorks™, X-10™, 0–10 V and DALI™, and proprietary protocols.

From the previously mentioned protocols, the system described in this work uses protocol IEEE 802.15.4 for wireless communications and the DALI protocol to control the lamps. The Digital Addressable Lighting Interface (DALI) is designed to digitally control electronic ballasts and luminaries equipped with this technology [11]. The interface and protocol are defined by standard IEC 60929/EN 60929 Section E.4, and were modified by IEC 62386, which also includes other applications for DALI besides lighting and expands the types of lamps to *high-intensity discharge* (HID) lamps, halogen lamps, incandescent bulbs, LEDs, *etc*.

The paper is organized as follows: Section 2 provides a short review of the state of the art in lighting control systems; Section 3 presents an overview on the architecture of the system; Section 4 describes the hardware and software implementation of the wireless node; Section 5 shows the results of some of the implemented tests and installations; and finally, Section 6 provides conclusions.

## 2. Related Works

Many works deal with the smart control of outdoor lighting systems to solve specific problems. On the one hand, they do so by using new technologies such as LED technology. On the other hand, the most innovative approach may be to use a remote control based on wireless communication and on control over smart ballasts. Several scientific studies have taken advantage of WSNs for public lighting systems. A ZigBee-based wireless control system is presented in [12]. Its aim is to overcome the disadvantages of a control group base by designing a public lighting control system that is able to control each luminaire individually. This study considers communication systems such as those based on power line communication (PLC) and ZigBee, and describe and compare their features. Finally, ZigBee is chosen due to the inconveniences of PLC, such as the price of the devices and the lack of communication when a short-circuit problem appears. Its system allows the user to control and monitor the state of the lighting; however, energy efficiency is not taken into account. Just maintenance and the removal of cables from the streets are taken into account to protect individuals from electric shocks.

Another more complex example of the application of WSNs to public lighting is found in [13]. A system is developed that consists of sensor nodes placed at each side of the street, with a receiver node at the main transformation station to which each luminaire belongs. Information from the receiver nodes is sent to the control center through GPRS. The system also has individual or group regulation, which saves up to 60% in energy consumption. A significant breakthrough of these systems is the combined use of identification addresses for the individual nodes, together with a geographic information system (GIS) that enables the graphic viewing of the location of any individual node. As a result, it is possible to know the location and state of each lamp. A simple but effective routing protocol is proposed in a later study [14] that makes it unnecessary to take the network topology into account, which is convenient due to the characteristics of public lighting.

Another proposal [15] lists the main characteristics of a WSN that may be used in a control system for public lighting. Among these characteristics, we find the ability to manage common operations in public lighting, such as turning off/on or regulation (dimming) and network functions such as neighbor discovery, pairing, and network formation. 6LoWPAN™ is used instead of ZigBee due to the disadvantages of the latter’s routing protocol (high end-to-end delay, low packet delivery ratio) and the easy adaptation of 6LoWPAN, which does not define the routing protocols for a specific system.

Other more current examples of the application of WSN to public lighting can be found in [16], in which the authors analyze the use of solar energy and propose a stand-alone streetlight system using a ZigBee network supplied by solar PV panels. Using part of the previous work, in [17], the authors present a fully controlled street lighting “*isle*” with a WIMAX connection. Lastly, we point out [18], which presents a streetlight control system based on wireless communication using LED technology. However, the authors do not use any standard protocol to control the lamps.

Other perspectives have been considered for the use of the DALI protocol in public lighting and its integration with WSNs. In fact, the NumeLiTe Project [19,20] used the DALI protocol for street lighting applications, but the manufacturers did not take this into account and the project was finally used just for indoor lighting and not for public lighting systems. Another work developed a DALI-protocol-based electronic ballast for sending commands through radio waves with a ZigBee module [21].

## 3. System Architecture

**Figure 1:**
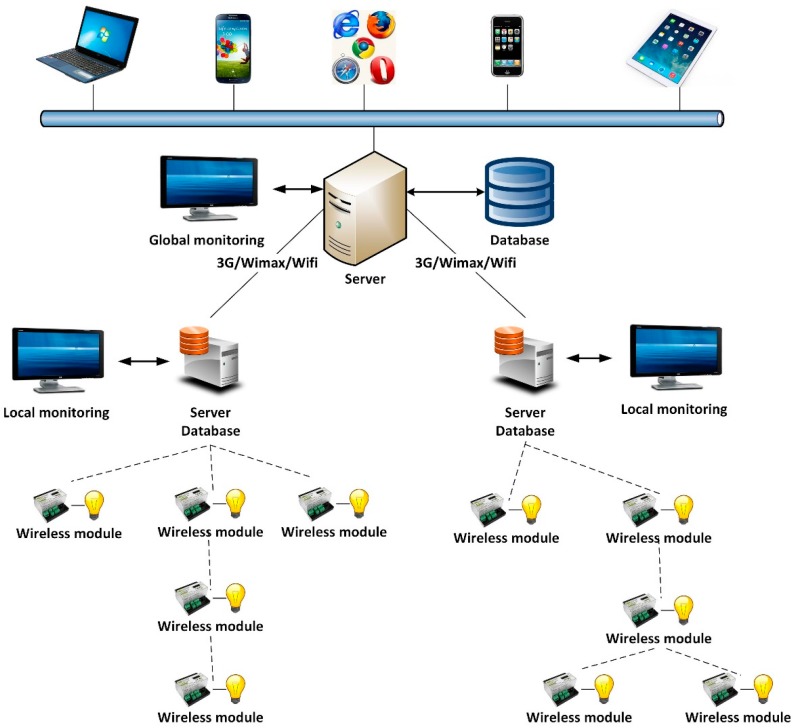
Architecture of the system.

Figure 1 shows the general structure of the tele-management lighting system described in this work. It is a three-level hierarchical model that is widely used in lighting-control systems based on wireless communication. To complete the system, an information and failure location application was developed for mobile devices. This app can be easily used by maintenance technicians.

At the lowest level, directly controlling the luminaire, we can find the wireless nodes, which communicate in a wireless way using IEEE 802.15.4. At the intermediate level, managing the wireless networks of a lighting sector, we can find an industrial computer with 802.15.4 communication that also acts as the network’s coordinator. This computer contains an application that enables the comfortable and user-friendly management of a given sector’s lighting network. This local computer can also act as a gateway towards central control of the lighting network. The communication with the central control is executed through another communication network that allows for greater distances, such as 3G/GPRS, WIFI or WIMAX. Lastly, the upper level contains the central control, which groups several sectors of the lighting network.

The local monitoring of a lighting sector is essential, as the central control may be very far away or have obstacles exceeding the physical capacities of the wireless protocol. On the other hand, the local computer for each lighting sector can work in isolation, since it can work as a server database. This characteristic ensures easy deployment and scalability of the telemanagement system. New sectors of the lighting network that are added to the central control can be debugged. Then, a more detailed description of each part will be given.

### 3.1. Network Topology

**Figure 2:**
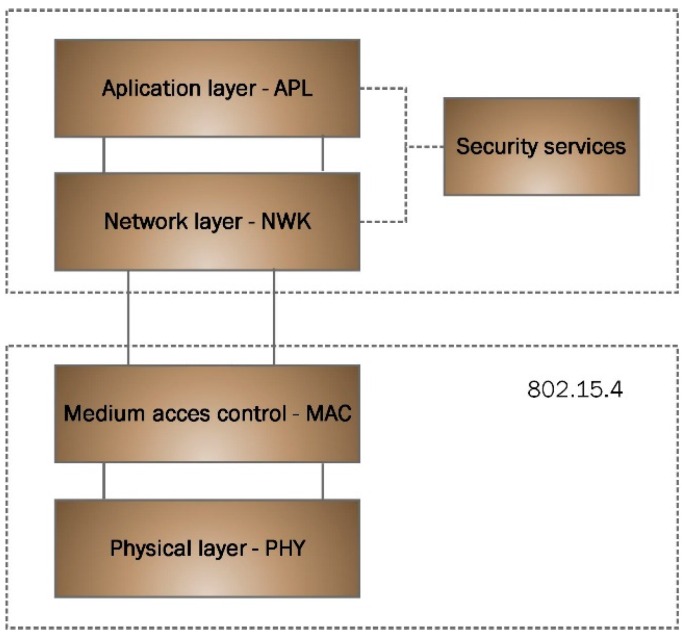
Layers model.

The 802.15.4 standard is framed within the area of the wireless personal area networks (WPANs), with low consumption and cost. As a result, it is increasingly gaining relevance in the industrial domain. Standard 802.15.4 only defines the physical and media access control layers. A network and application layer has been developed on this standard, as shown by Figure 2.

Standard 802.15.4 defines two types of topologies: star and peer-to-peer networks. The peer-to-peer topology allows each node of the network to communicate with any other node, provided that the node is within its range. The star topology allows the establishment of communication among the devices and of a sole central node that acts as the network coordinator. The network coordinator is responsible for starting and finishing the connections, and any communication between two nodes must compulsorily pass through the network coordinator.

The network layer must take the topology of the lighting network into account. On the one hand, each luminaire is controlled by a node with 802.15.4 communication capacities. On the other hand, the luminaires are often deployed along long streets, which results in a linear structure with slight branches. Taking this topology formed by the luminaires into account, a tree topology created from the peer-to-peer connection possibilities of standard 802.15.4 has been chosen.

In the tree topology, the coordinator is located on the first level of the network, with several end devices and a router. The router forms a new network level consisting of several end devices and a new router, and so on until the desired number of levels required in each case is achieved. The number of leaps allowed by the network layer must be high. This type of structure does not adapt well to any usual topology in wireless networks or to any standard. For example, Zigbee Pro has a limited tree-type number of network layers, with a maximum depth of 15. The number of network hops allowable within ZigBee PRO is increased to 30 from the 10 allowed under ZigBee.

A tree topology with a high depth entails a serious routing problem, as almost all nodes need to have this ability. In [22], a novel routing algorithm is proposed for streetlight systems. Additionally, there are few alternatives in the event of an intermediate node failure. The allocation of addresses turns into a complex problem as well. In [23,24,25], a new address-allocation method that improves ZigBee tree addressing is proposed, which offers some results in simulations. For these reasons, we decided to create our own network layer that could adapt to a tree topology with a high number of levels: the computer that serves as the origin or destination of all exchanged information on the network. That is, we have implemented the communication by using IEEE 802.15.4 Std. and by creating our own (ad-hoc) Network layer (NWK).

Each node has two unique identifiers—the radio module’s MAC address and a node identifier (nodeID)—that allow it to be located by the application, similar to a lamp address. In this way, when a radio module is replaced, only the same nodeID needs to be selected in the node. This nodeID is easily set with a microswitch. The nodeID has a length of 16 bits and can select up to 65,536 ballasts, which increases the number of ballasts that can be routed by a DALI controller. The user application only needs to know the nodeID. The network layer maintains some tables with the MAC addresses where the frames need to be sent to get to their destination. These tables are dynamically created when the network is initialised, when a node is connected, *etc*.

### 3.2. Lighting Control: DALI

Most current digital ballasts implement the DALI protocol. Additionally, it is also implemented in low-voltage transformers, movement detectors, wall switches, *etc*.

The main reason for choosing DALI over other lighting control protocols is that it allows for bidirectional communication (unlike X-10, 0-10 V and DMX). On the other hand, its physical interface is very simple, as it uses only two wires for the control signals. Thanks to the use of this protocol, the following advantages are achieved:

- Failure detection for lamps or electric failures
- The ability to take and send data related to the lamp’s status
- Level dimming
- Better energy consumption
- Information on the lamp’s voltage, current and power factor
- The ability to execute other actions, such as turning the lights on/off, controlling them, *etc*.

**Figure 3:**
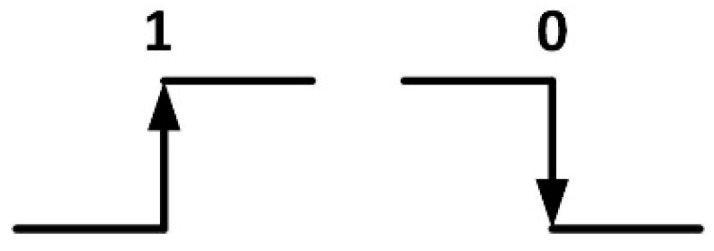
Differential Manchester encoding.

The physical layer of the DALI protocol uses Differential Manchester encoding, in which the logic values are represented by transitions in the signal, as shown by Figure 3. Each node is attached to the bus line via its two bus terminals, which provide differential receiving and transmitting capability.

**Figure 4:**
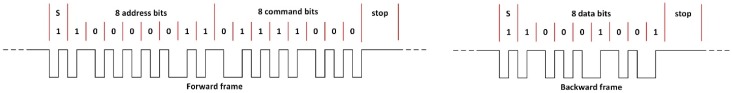
Format of the DALI frames.

The DALI protocol is based on the master/slave model, in which one master can control up to 64 slaves. The master sends frames (forward messages) to any slave device of the system and receives a response frame (backward message) if the forward frame requested a response. As shown by Figure 4, a forward master–slave frame (ballast) consists of 19 bits, distributed as follows: one starting bit, eight bits for routing, another eight bits for data and two stop bits. Meanwhile, a backward response frame consists of 11 bits, a start bit, 8 bits for data and two stop bits. At the physical layer, DALI uses an effective data transfer rate of 1.2 kb/s and a tolerance in the timing bit of 10%.

The main disadvantage of the DALI protocol is that it is not implemented in most microcontrollers. In addition, the logic levels of the physical layer have incompatible voltage values with most microcontroller-based systems.

### 3.3. Wireless Node

An important aspect of developing streetlight control systems is deciding which components to develop and which ones to purchase from the market. To make this decision, it is important to study whether these components exist in the market, their price and the economic impact on the project, among other factors. In the presented system, we have to pay special attention to the wireless node (mote), as a mote needs to be installed in each luminaire. As a result, a high number of motes are needed. A comparison of several motes was implemented in [26], and in our case, several commercial motes such as Waspmote [27] have been analyzed, in particular, together with other open-source, Arduino- and hardware-based nodes [28]. All of them show the following disadvantages in our system:

- They do not have a DALI interface.
- They are not properly powered. The input voltage is 230 VAC; hence, the voltages for the processing part and for the DALI interface must be generated, as explained below.
- The nodeID cannot be configured in an easy way by maintenance technicians if a mote needs to be replaced.
- No robust connectors are available.

We decided to design our own mote, since if we had opted to use commercial mote, we would have needed to design an adaptation board to solve the aforementioned disadvantages. The designed mote has two different parts: the processing and radio module, and the rest of interfaces, including the DALI interface and the power supply block. The reason for this division is to try different radio modules, so that both parts can later be integrated into just one board. In this way, we would obtain a specific mote to control the ballasts at a low economic cost. The designed mote will be described in detail in Section 4.

The decision to design a specific mote is a usual process in many works related to wireless sensor networks. In works such as [29,30,31,32], motes were developed for specific applications to minimize total consumption. In [33], a wireless sensor mote was designed for precision horticulture applications. In other works, some motes were designed to search for possibilities not offered by commercial motes. For example, in [34], a mote was designed to support remote hardware modification. Lastly, regarding wireless nodes to control LED lamps in over-the-air lighting-control systems, motes using the XBee module of the Digi MaxStream radio frequency were designed in [16,35]. In [18], the Waspmote module was improved by connecting sensors to it and including control over LED lamps. In [36], a simple intelligent wireless module was presented for use in street lighting systems that provides a specific driver capable of communicating through the DALI protocol to the lamp.

### 3.4. Supervisory Control and Data Acquisition System

A SCADA (supervisory control and data acquisition) system was developed to control lighting systems. This application is executed on an embedded industrial computer in each wireless network of a section of the lighting network. The computer communicates directly with the coordinator node of the wireless network. Another SCADA system has been planned to control more than one section of the lighting network. This central SCADA system communicates with each local SCADA system. The central SCADA system collects the information from the local SCADA. The local SCADA is actually the one in control; as a result, it will be the one described below.

The software used for the execution of the SCADA is the integrated development environment (IDE) NetBeans [37]. NetBeans is a free product without any restrictions on its use. It was mainly designed to be used with the Java programming language. In this way, it can be used in any Java virtual machine (JVM), no matter the architecture of the computer used.

**Figure 5:**
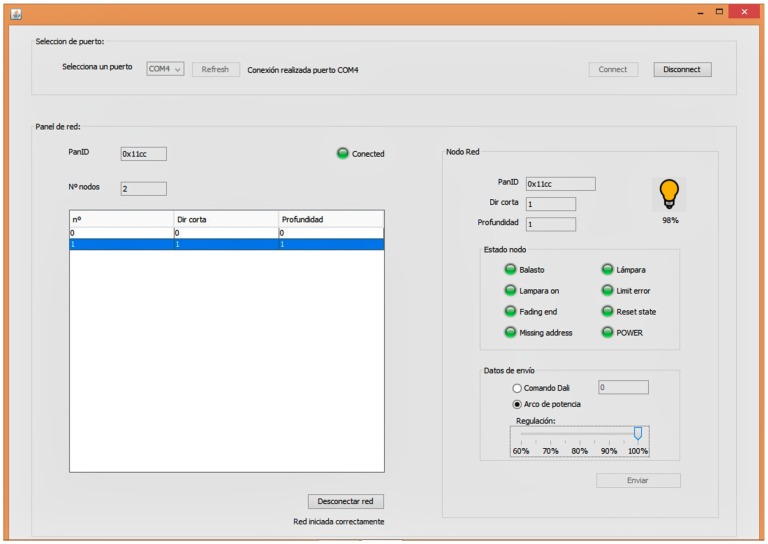
View of one of the windows of the local SCADA.

By means of SCADA, a user with the appropriate licenses has both individual and collective control over the lamps. The coordinator (who is connected to the computer) deals with forming/managing the tree wireless network, so that an intermediate router node will be used to reach a lamp if the coordinator is unable to do so. Once all of the luminaires have been located, their status may be viewed and modified, making it also possible to specify different modes of operation from the interface. Figure 5 shows one of the windows of SCADA.

**Figure 6:**
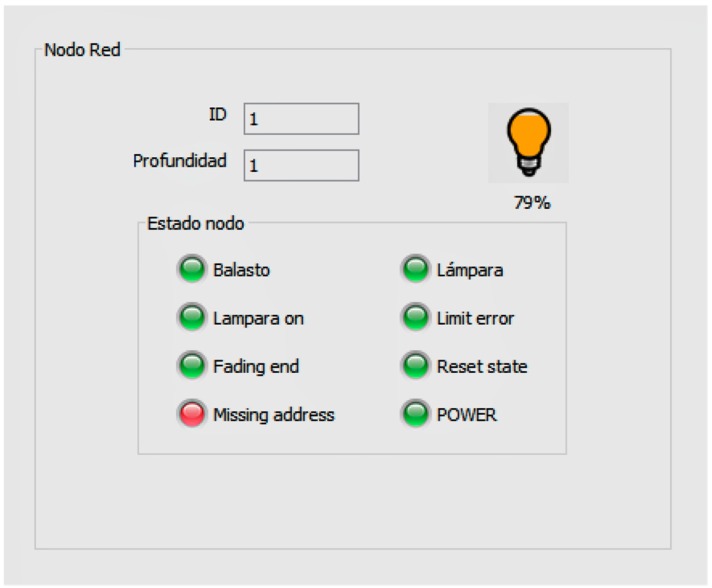
Detail of the information window of a lamp.

As an example, Figure 6 shows the information of a selected street lamp, which allows the user to know:

- Whether the light is switched on or off.
- Its current regulation setpoint.
- The existence of limit error events.
- The existence of lamp error events.
- The existence of ballast error events.
- The reset state.

### 3.5. Application for the Maintenance of the Lighting Network

**Figure 7:**
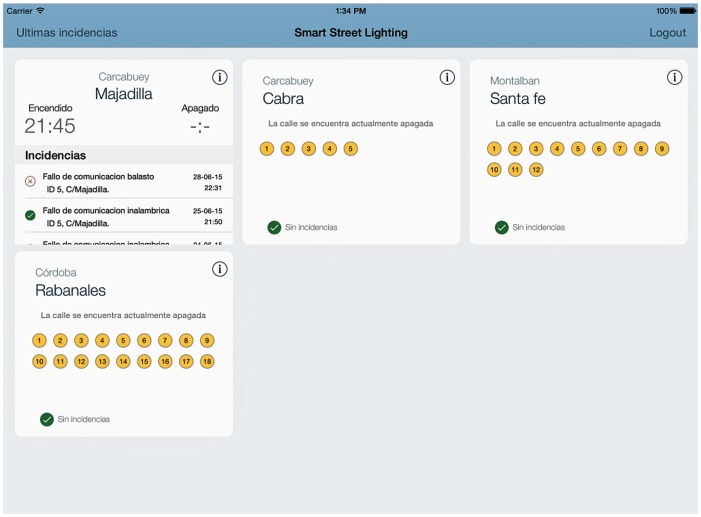
App screenshot with information on incidents for the maintenance technician.

A graphic application for mobile devices has been developed to manage maintenance tasks for lighting networks. The application obtains information from the database created by the SCADA system. Once the application has been launched and the user has been identified, the streets allocated to the operator will appear. If any failure has happened, the application will inform the user with an interface such as the one shown in Figure 7. Each streetlamp is identified by a unique identifier (nodeID), which is set by hardware when the node is installed, as mentioned in Section 3.1.

**Figure 8:**
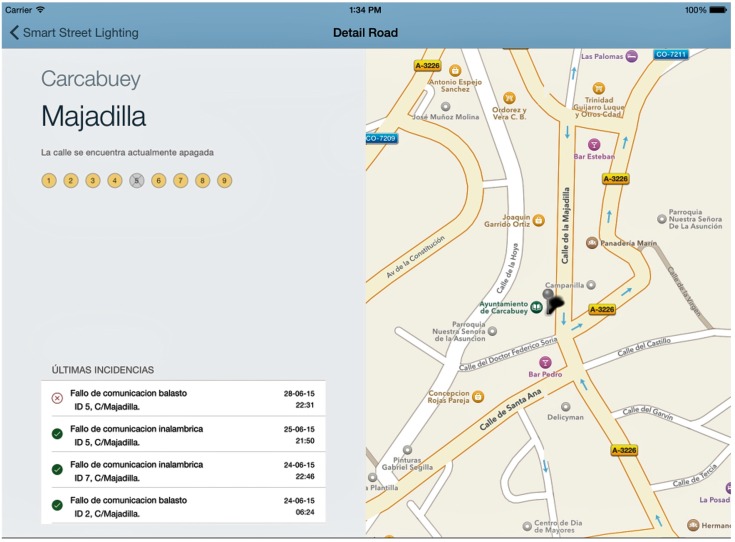
App screenshot GIS location of an incident.

From this moment, the application includes several screens, such as the one shown in Figure 8, which helps the technician to determine the location of the streetlight. This system is particularly useful in small towns or villages where just one company is responsible for maintaining the whole lighting system, preventing unnecessary driving around to control the status of the streetlamps.

This is the case, for example, in some areas of Andalusia (Spain), which has scattered small towns and villages where having an onsite maintenance technician is impossible. Once the technician has solved the problem, the system detects it and the database is updated accordingly.

## 4. Implementation of the Wireless Node

As was already discussed in Section 3.3, we designed our own wireless node for several reasons. We will describe in detail both the hardware and the software of the UCODALI wireless node in detail below.

### 4.1. Hardware Node: UCODALI Board

**Figure 9:**
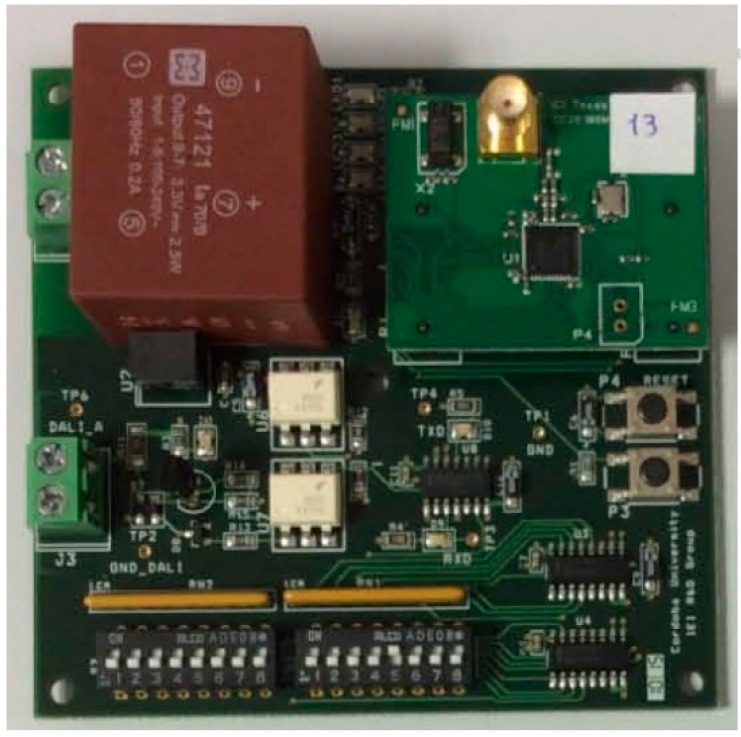
UCODALI version 2 board.

This section will include a description of the hardware of UCODALI board, version 2, shown in Figure 9.

**Figure 10:**
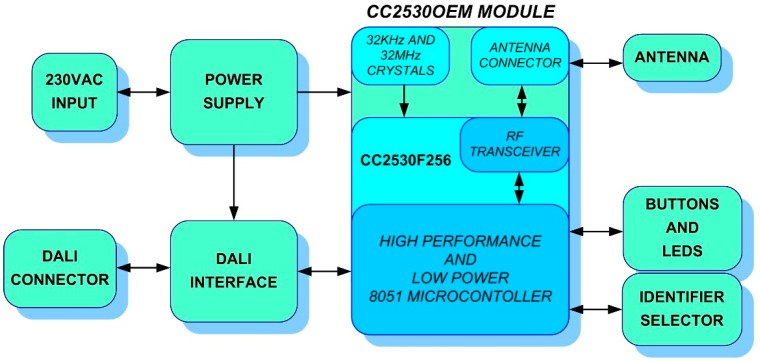
UCODALI block diagram.

Until now, two versions of the UCODALI board have been designed to implement the sensor network node with the DALI interface. Both versions are very similar. Its block diagram is shown in Figure 10, in which the following can be distinguished: the power supply, CC2530OEM module [38], DALI interface, selector of the nodeID and HMI (buttons and LEDs) blocks.

#### 4.1.1. Power Supply Block

All of the board’s components, except for the DALI interface block, need a power supply voltage of 3.3 VDC. We used the FC47121 switching power supply [39] to obtain this voltage from the 230 VAC network. This power supply has the following characteristics: 3.3 VDC output voltage, ±2% output voltage accuracy (100% load), 2.5 W power and 65% efficiency. This switching power supply is a switched mode power supply based on flyback topology that provides an alternative solution to traditional power supplies used in low-power applications.

As will be indicated below, the DALI interface uses voltage levels to represent the high logic level between 11.5 V and 22.5 V. As a result, a power supply that generates a voltage within this range needs to be used. On the other hand, the control part of the node of the DALI bus needs to be isolated. To do this, we used the MEU1S0315ZC DC/DC converter by Murata Power Solutions [40]. The MEU1S0315ZC is an ultra-miniature through-hole 1 W DC/DC converter that is available in a ZIP-style pinout. It is ideally suited for providing local power supplies on control system boards, with the added benefit of 1 kVDC galvanic isolation to reduce switching noise. From the 3.3 VDC voltage, it generates an output voltage of 15 VDC with 81% efficiency.

In the first version of the board, a FC47125 switching power supply was used instead of the MEU1S0315ZC DC/DC converter. The use of this converter and the fact that version 2 has 4 layers—compared with version 1, which had just 2 layers—have allowed us to reduce the size of the board by 40% and reduce its total cost.

#### 4.1.2. CC2530OEM Module

**Figure 11:**
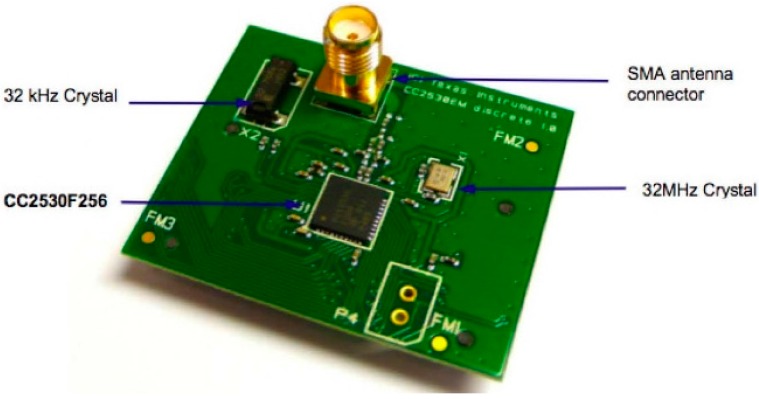
CC2530OEM module [Source: TI.com].

Figure 11 shows a photograph of the CC2530OEM module. This module is the main component of the board, as it acts as a processor, controls the node and implements both the DALI communication and IEEE 802.15.4 protocols as well as the necessary network and application layers for the remote management of lighting ballasts. Our research group selected this module because we have broad experience using Texas Instruments wireless modules and because its characteristics and features meet the requirements for our node.

It has reduced power consumption, although this is not an essential requirement here, as the node will be powered directly by the 230 VAC network. The module contains a CC2530F256 chip [41]; two quartz crystals, one 32 MHz and the other 32 KHz; an SMA antenna connector; and a radiofrequency impedance adaptation circuit. The CC2530F256 has, in just one chip, an IEEE 802.15.4-compliant RF transceiver, an 8051 microcontroller, an AES Security Coprocessor, flash memory and RAM.

The CPU 8051 enables the execution of an instruction period, as it has three different buses with which to access the SFR (special function register) memories, DATA (data register) and SRAM. On the other hand, having three general-purpose timers is very important for our objectives, for us to be able to implement the Manchester coding–decoding used in the DALI interface.

#### 4.1.3. DALI Interface

**Figure 12:**
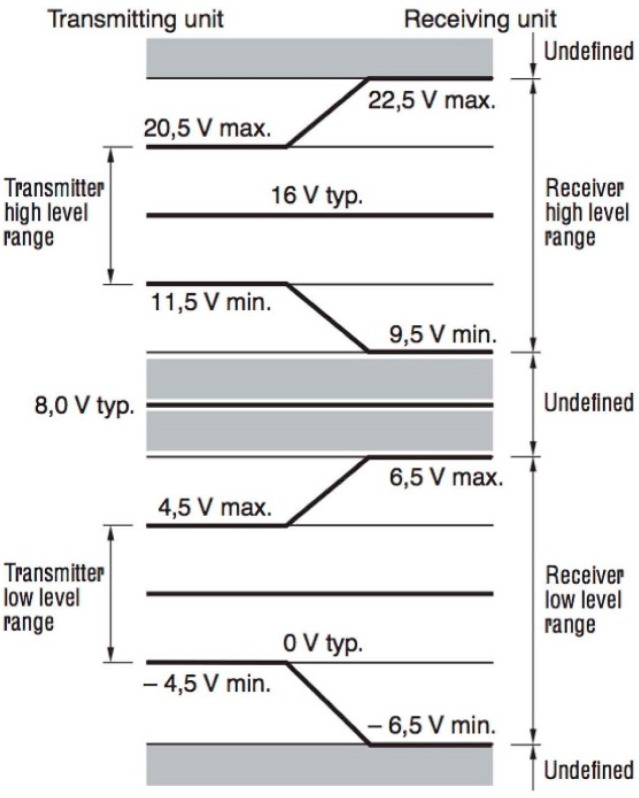
DALI logic levels.

The DALI bus consists of two wires that transmit information in a differential way. The maximum communication distance is 300 m. Logic levels are determined by the difference in voltage between both wires of the bus. The logic levels of the DALI control interface range from 9.5 V to 22.5 V for the high logic level, with the ballast acting as a receiver, and 11.5 V to 20.5 V when acting as transmitter. Meanwhile, on a low logic level, they vary between −4.5 V and +4.5 V for the transmitter and between −6.5 V and +6.5 V for the receiver, as can be seen in Figure 12. Because the logic levels of DALI are not compatible with those of the CC2530 control module and because both need to be electrically isolated, we designed an interface that meets both requirements.

Even though several manufacturers have developed several types of microcontroller/DALI interfaces, such as Texas Instruments’ MSP40 [42], we have decided to design our own interface because the interface from TI does not feature a DALI bus power supply and is not protected against polarity changes.

**Figure 13:**
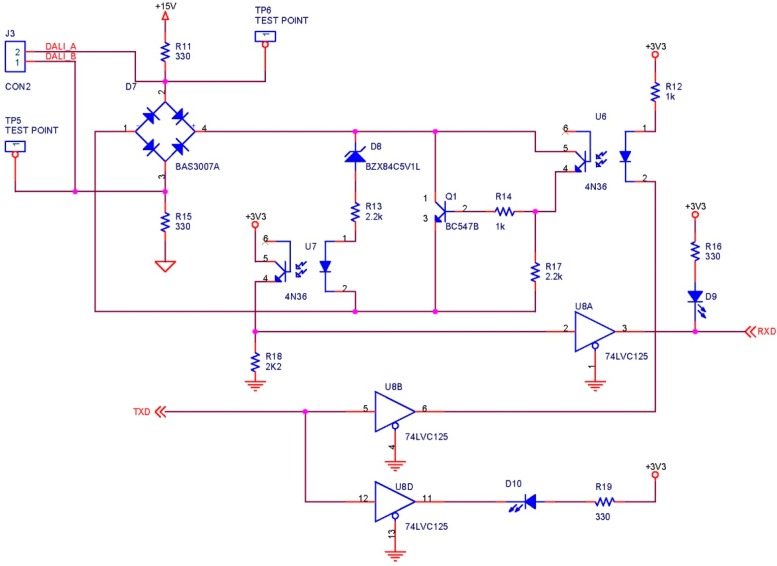
DALI interface.

Figure 13 shows the designed DALI interface. In its design, we took into account that it must support bidirectional communication. We used two optocouplers with 4N36 phototransistor outputs to isolate both the transmission and the reception of the DALI bus microcontroller. It was designed for a low logic level in microcontroller’s pin to correspond to a low logic level in the DALI bus, and in the same way for a high logic level. In this way, the bits of the frames should not be complemented by software, either in transmission or in reception. The diode bridge is included in the connection to the DALI bus to protect against polarity changes, in case further ballasts are connected and an external source is used. This bridge is a Schottky diode to ensure a low forward voltage drop and a fast switching speed.

The operation is very simple. If the microcontroller sets the transmission signal (TXD) to a high logic level, the optocoupler diode U6 does not conduct. As a result, its output phototransistor will be cut off, just like the Q1 transistor. In this way, the voltage in the DALI bus will be 15 V minus the voltage drops in the diode and the resistances, obtaining a final value of 11.5 V, according to the implemented tests. As a result, the DALI bus will have a high logic level. Oppositely, if the microcontroller sets the TXD to a low logic level, the U6 optocoupler diode will conduct and will saturate its output phototransistor. In this way, the Q1 base will receive enough current to be driven and, due to its high current gain (200 min.), to establish a voltage of 1.5 V maximum in the DALI bus, *i.e.*, a low logic level. By using the Q1 transistor to control the DALI bus, a low current will circulate though the optocoupler diode when it is conducting. In this way, the reliability of the circuit will increase.

The operation of reception is very similar. If the ballast establishes a high logic level in the DALI bus (9.5 V minimum), it will switch on the diode bridge, the Zener diode and the U7 optocoupler diode. As a result, its output phototransistor will saturate and the reception signal (RXD) will be set to a high logic level. If the ballast establishes a low logic level in the DALI bus (6.5 V maximum), the voltage will be insufficient to bias the diode bridge, the zener diode and the U7 optocoupler diode. As a result, its output phototransistor will be cut off and the RXD signal will be set on a low logic level through the R18 resistance.

**Figure 14:**
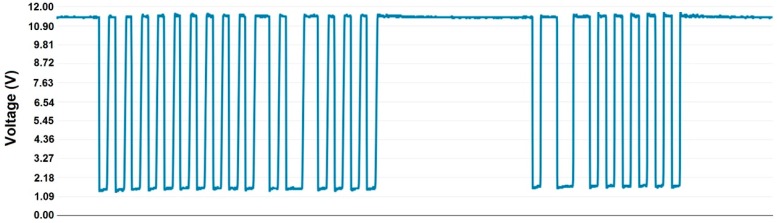
DALI frames.

Figure 14 shows a DALI frame for transmission/reception. The first 19-bit frame corresponds to a forward frame sent by the microcontroller to the ballast. The second one is the response frame of the ballast (11 bits). As can be seen, appropriate DALI levels are obtained. The signal is set to 11.5 V at the high logic level and to 1.2 V at the low logic level.

#### 4.1.4. NodeID Selector

This block consists of two 8 encapsulated microswitches and two 8-to-1 multiplexers. A 16-bit node identifier is set by means of the microswitches. In this way, if an action event on a streetlight takes place, this event will be easily identified by the technician. Due to the limited number of I/O pins on the CC2530OEM module, the address is read through two 8-to-1 multiplexers, one for each microswitch. In this way, just three signals are needed to control both multiplexers at the same time, plus two to read the data. Once the data have been read, the application will be able to process them correctly and obtain the 16-bit identifier (nodeID).

#### 4.1.5. Push Button and LEDs

The board contains two push buttons. One of them is the Reset button, which the technician can push if the node is in any anomalous state for any reason. The other button was included for manual tests, so that the node connects to the coordinator when this button is pushed.

On the other hand, some LEDs have been included to monitor the power supply’s voltages, the transmission and reception signals of the DALI bus with the microcontroller and the state of the node. The state LEDs show the technician the state of the node, such as if the wireless node is scanning or if it has been associated with another node.

### 4.2. Software Node

As mentioned in Section 4.1, we used a processing and radio module: the SOC 2530 by Texas Instruments. To develop wireless applications with this circuit, Texas Instruments provides the IEEE 802.15.4 medium access control (MAC) software stack TIMAC [43] for free. ZigBee was built on top of this standard. TIMAC consists of three components: MAC (IEEE 802.15.4 Medium Access Control Software Stack), HAL (Hardware Abstraction Library) and OSAL (Operating System Abstraction Library) [43,44,45]. IAR Embedded Workbench EW8051 [46] was used as the development environment.

**Figure 15:**
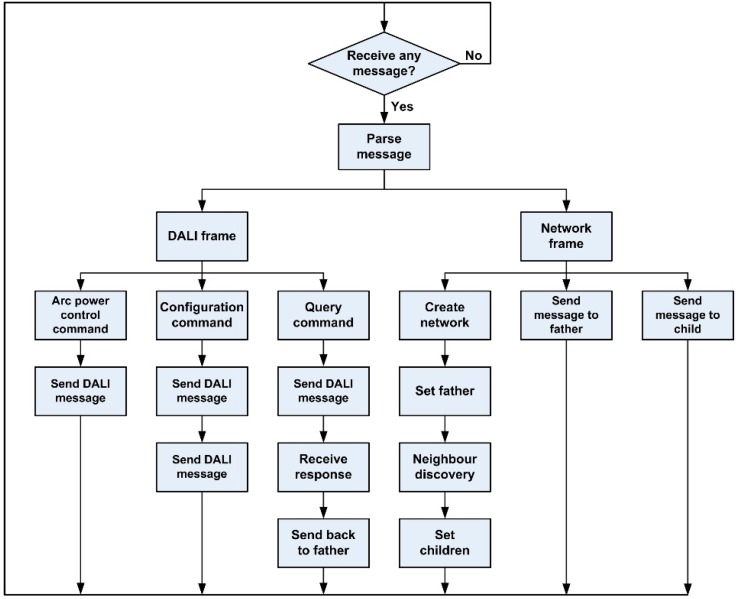
Microcontroller program flowchart.

We created an application and a network layer on the medium access control layer for the wireless nodes. The application network is responsible for analyzing the received frames and for acting on the lamps and the rest of the node’s peripherals. The network layer is responsible for the network creation operations and for appropriately routing the packets. Each packet that arrives at the node is processed to check whether the node is its final destination. Otherwise, it is routed towards another node. The node reacts depending on whether the received message is a DALI message (taking into consideration the kind of DALI message received) or a network message, according to the flowchart shown in Figure 15.

The RF module only supports point-to-point and star (point-to-multipoint) communications. In a street lighting system, it is essential that the PAN coordinator can reach any node. This requirement makes the star topology completely useless, so we were required to modify the network layer somewhat to ensure a tree topology. The nodes have several tables containing the routing information of the messages. When the network is created, each node also creates a table where the nodeID is related to its MAC address or to the MAC address of the node to which the messages destined for the nodeID need to be routed.

The coordinator’s table contains the MAC address of the node and every router in the way of the node. The table size is predefined by the total length (maximum depth) of the network. Another field of this table is the child number that the node represents for its father. The table stored in any node only contains the MAC addresses of its children, in an order given by the coordinator. This way of creating the network allows the coordinator to use source routing packet transmission, in which the coordinator puts in the packet the addresses of the nodes through which the message must pass.

From the individual node point of view, there are only two kinds of DALI frames, according to whether they require a response or not. For instance, for an arc power control command, just a frame is transmitted without any response from the ballast. In contrast, a backward frame is received for a ballast status query command.

The microcontroller also manages the conversion to/from the Differential Manchester code. The microcontrollers do not usually have an integrated peripheral that communicates using Manchester coding, nor does this peripheral implement the DALI protocol. We needed to create this component using software and by creating a set of functions for the user to use to communicate with this virtual peripheral. The software component for DALI communication is relatively complex, as it must await responses from the DALI slave, consumes three timers of the microcontroller and is implemented on a physical level with input/output port pins.

**Figure 16:**
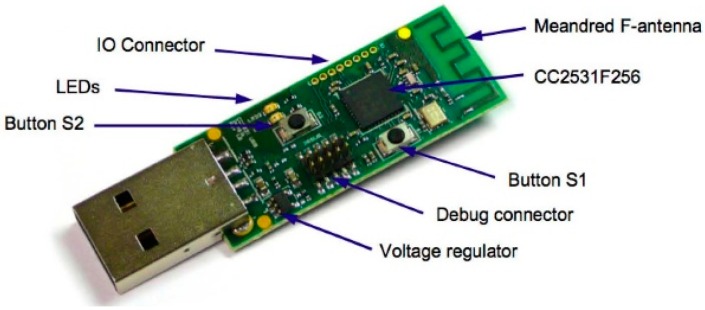
CC2531 USB Dongle [Source: TI.com].

The coordinator node communicates directly with the industrial computer through USB and does not control any lamp. Because of this, we used the CC2531 module by Texas Instruments, shown in Figure 16 [47]. In any case, any other module identified by the computer as a UART (universal asynchronous receiver-transmitter) can also be used.

Lastly, we took into account the fact that the wireless modules installed in the street lamps are within anyone’s reach. Because of this, some encrypted frames using a SOC CC2530 security coprocessor with 128-bit AES encryption and pre-shared key authentication have been used.

## 5. Experimental Results: Test Scenarios

### 5.1. Lab Test

Before the system was installed in any given location, some experiments were implemented in the lab, where it is easier to check the system’s functionality. The nodes were placed in separated labs to have the walls limit the radio range. In particular, we implemented connection and reconnection tests for the nodes and control of the lamps.

**Figure 17:**
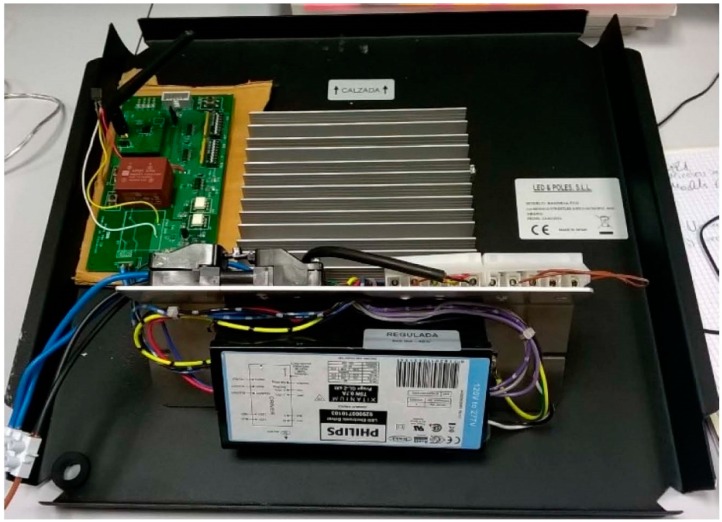
System used in the lab to control a 46 W LED lamp.

We executed a global operation test that saw the whole system as a black box. The global test consisted of programming some lamps in the SCADA system with a given regulation and watching the power consumed by each lamp. We implemented tests with different lamps to check that the dimming by the designed telemanagement module was working correctly. A high-pressure 100 W OSRAM VIALOX NAV(SON)-E sodium vapor lamp and a LED luminaire were used. The tests implemented with the LED luminaire will be just commented upon as an example. In particular, the BANDEJA FCO model by LED & POLES S.L.L. with STREETLED ASW/X-50/740 IP65 46 W module with adjustable Xitanium 75 W 0.7 A Prog + GL-Z sXt module by Philips was tested. Figure 17 shows an image of the system consisting of the LED luminaire, the Xitanium driver and the UCODALI board.

**Figure 18:**
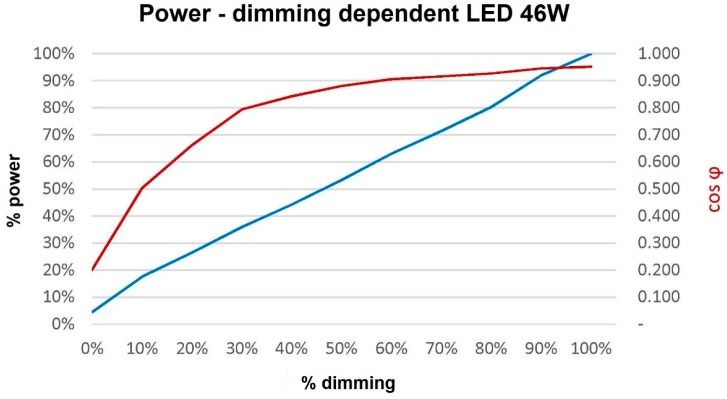
Power curve and cos φ depending on the dimming.

We measured the active power, reactive power, apparent power and cos φ of the LED lamp. To do so, we used a California Instruments 3001 IX [48] power supply, connected to a data acquisition board by National Instruments that was inserted into a PC, and a user interface by means of the LabView tool. Table 1 and Figure 18 show the results of these measurements.

As a result, these data show that the power dimming of the lamp controlled by the remote management module works properly. Thus, it also proves the good communication between both through the DALI bus.

Regarding the network tests, nodes have been connected and reconnected to check if the network was correctly allocating the addresses and if the SCADA system informed the user about the connection and reconnection in an automatic way. The lighting network has also been tested regarding turning on/off without fulfilling the communication with all of the nodes to check whether the lamps begin the previously established operation curve when there is no communication with the SCADA system.

### 5.2. Onsite Test

The lighting control system has been installed in two towns within the province of Cordoba, in the Andalusia region in southern Spain. In both cases, the system was deployed without any incidents and is currently being used. Before the deployment of the system, a photometric study was performed to determine the correct location of the lamps.

**Figure 19:**
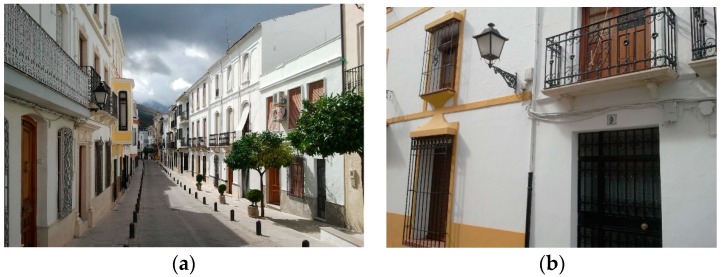
(**a**) General overview of the street; (**b**) Details of the lamp post installation.

Figure 19 shows, in detail, the type of luminaries installed in the city centers of both towns. Thanks to the wireless communication, no new wiring was necessary. The network created is a linear network with a depth of 7. The coordinator node, together with the embedded computer, is located inside of the town hall, at the end of street.

**Figure 20:**
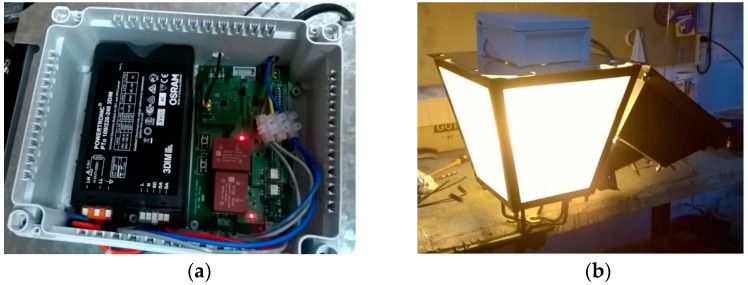
(**a**) Wireless node and ballast; (**b**) Installation of the node into the luminaire.

Figure 20a shows the connection and placement of the wireless node with the ballast, whereas Figure 20b shows the location of the system in the luminaires. The RF antenna is located on the outer side of the luminaire.

**Figure 21:**
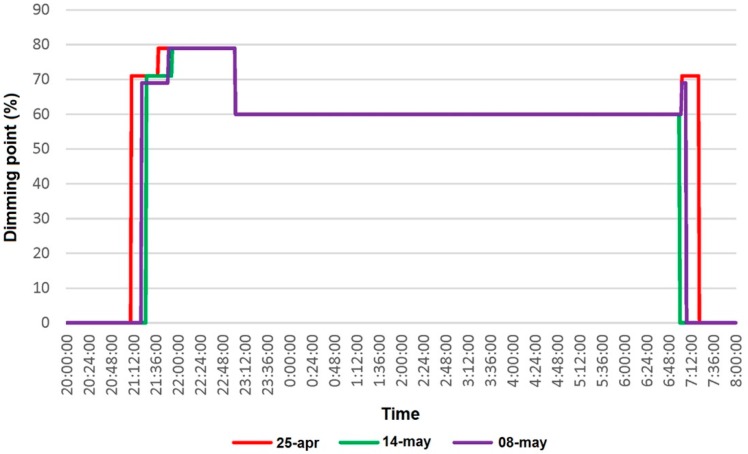
Data collected by the SCADA system (lamp #1).

As an example, Figure 21 shows a graph with real data obtained during three different days (24–25 April: red, 13–14 May: green and 7–8 May: purple) from a lamp installed at the street. The running sequence is as follows:

- Lights are turned on later every day due to the daily delay of the sunset. At sunset the lamp is turning on at 70% set point (smooth turn-on and warming period).
- 30 min later the set point is set at 80% (full illumination set point).
- At 11:00 p.m. the dimming is reduced to 60% until 7 a.m. of the following day (saving mode operation).
- At 7 a.m. is set again at 70% until sunrise (sunrise turn-off mode).

The smooth turn-on and warming period is performed to optimize their lifespan and reduce peaks, flickers and other instabilities that used to appear in the main grid when switching on a huge number of lamps. After the smooth turn-on period the dimming increases to turn into full illumination. Later the dimming is set to the saving mode operation. From that moment onwards when starts the sunrise turn-off mode (turns again to 70% until sunrise when are turned-off). As can be seen, on 14 May, these lamps did not reached the sunrise turn-off mode as the sunrise happened before 7 a.m.

The most common control modes for streetlighting that can be found are those based on controlling the on-off timespan of the lighting using astronomical time relays (ATRs). In this manner, the lamp will be switched on at 100% at sunset and will switched off when sunrise, *i.e.*, the lamp will be at 100% during its entire operation time. Another very common control mode is the last one combined with double-level ballast (DLB), by which the lamp starts lighting at 100% and drops to 70% several hours later (defined manually by microswitches by the technical operator) [49].

The proposed system clearly presents advantages *versus* the others mentioned, both in technical aspects as well as in economic and environmental ones. The energy savings that can be extracted from Figure 21 are about 30%–40% of the overall operation time of each lamp as shown in Table 2, *versus* the ATR or DLB modes, and comply with lighting level regulations.

The savings can be even higher because the lighting levels at 60% of the setpoint are still higher than the threshold established by regulations. In addition, the remote management interface enables the lighting schedule to be changed in real time to easily adapt the lighting levels and timing to needs, in an easier and more powerful way than the aforementioned existing methods.

Once proven that the proposed system runs as expected and using the experimental data gathered a simulation of the expected behavior for a whole year (January to December) have been done for the onsite installation mentioned. The results are shown in Table 3 and Table 4.

In terms of energy consumption and energy cost, the proposed system present clear savings *versus* others (ATR up to 31% and DBL up to 5%). Regarding price difference: it is constantly decreasing. Last year the difference was about 10%—regarding a luminaire alone (UCODALI or DBL *vs.* ATR). If the replacement regards a pole and luminaire, it drops to about 3%–4% (UCODALI *vs.* DBL). We are aware that the prices vary, depending heavily on particular market properties. That is why we are focusing on energy savings. Calculating Total Cost of Ownership and ROI requires further research and comparative market analysis.

From a technical point of view, this system allows users to fully customize the set of dimming setpoints and the switching time, *versus* the fixed and limited sets provided by the ATR and DLB methods. In addition, the presented system optimizes the lamps’ lifespan and provides a very valuable tool to the end user (mainly public administrators, e.g., citizens) for maintaining street-lighting facilities because the bidirectional communication data flow gathers relevant information about the status of the lamps and provides a source of information to detect any failure or malfunction in the luminaries.

Compared to other existing methods and technologies, like those based on power line communication (PLC) or LONWorks™, the system presents a clear advantage due to its wireless nature. No additional wires or installations are needed other than attaching a UCODALI board to the ballast into the luminaire. This obviously represents a huge benefit in terms of time and money when renewing existing facilities, acting in historical areas or buildings and even for new infrastructure.

From economic and environmental points of view, the benefits are clear in terms of energy savings: less consumption, lower costs and less CO_2_ emissions, in addition to the benefits obtained from optimizing the lifespan and maintenance of the lighting.

## 6. Conclusions

We presented a lighting control system based on wireless communication with a DALI interface. The system uses a wireless communication based on standard IEEE 802.15.4 over which a new network layer adapted to a linear topology has been created.

We described the architecture of the system, which is based on a three-level hierarchical model, and developed a SCADA application that allows the lamps’ operation curves to be controlled individually. This application also detects possible failures in the lamps, which are immediately communicated using cloud architecture thanks to a developed mobile application.

We developed a new low-cost wireless node with a DALI interface. This node is powered from mains (230 VAC), which makes it easily installable beside the lamp. The node can drive any DALI ballast or driver; it works with any kind of lamp, from high-pressure sodium lamps to LED lamps. In addition, due to the use of WSNs, the number of ballasts that can be managed with just one DALI controller has been increased.

The implemented tests and a brief description of the pilot installations have been presented. We have also presented and discussed experimental results that provide evidence that the system performs correctly.

The fully customizable time-dimming operation provides clear savings in terms of energy consumption, which means benefits in terms of environmental aspects (less CO_2_ emissions) plus profit due to lower energy costs. In addition, the system optimizes the lamps’ lifespan and provides a very valuable tool for maintaining street-lighting facilities because the bidirectional communication enables relevant information to be gathered about the status of the lamp and provides information about events, failures or malfunctions in the luminaries. Besides, it gives additional benefits being able to integrate lighting network with other Smart City solutions increasing safety and quality of life.

## Figures and Tables

**Table 1:** Consumption and illuminance values at three checkpoints after programming.

Dimming	Flow	S (VA)	P (W)	Q (VAr)	cos φ
0%	0%	9.8	2.1	9.6	0.214
10%	10%	16.5	8.3	14.4	0.503
20%	27%	18.9	12.5	13.9	0.661
30%	35%	21.4	17.0	13.1	0.794
40%	45%	24.8	20.9	13.3	0.843
50%	58%	28.6	25.2	14.0	0.881
60%	69%	32.9	29.8	14.4	0.906
70%	76%	36.9	33.8	14.6	0.916
80%	85%	41.0	38.0	15.0	0.927
90%	91%	46.0	43.5	15.5	0.946
100%	100%	49.7	47.3	15.5	0.952

**Table 2:** Measured consumption: energy, cost and saving rate.

Dimming		25-April	26-April	27-April	28-April	29-April	30-April	1-May	3-May	4-May	8-May	9-May	10-May	11-May	12-May	13-May	14-May
70% (h)		0.50	0.48	0.50	0.50	0.48	0.48	0.48	0.48	0.48	0.48	0.50	0.50	0.50	0.48	0.47	0.47
80% (h)		1.40	1.38	1.37	1.33	1.30	1.30	1.27	1.25	1.23	1.20	1.18	1.17	1.17	1.13	1.13	1.13
60% (h)		8.00	8.00	8.00	8.00	8.00	8.00	8.00	8.00	8.00	8.00	8.00	8.00	8.00	8.02	7.97	7.97
70% (h)		0.30	0.32	0.25	0.20	0.23	0.15	0.10	0.12	0.05	0.08	0.00	0.02	0.02	0.00	0.00	0.00
TOTAL (h)		10.20	10.18	10.12	10.03	10.02	9.93	9.85	9.85	9.77	9.77	9.68	9.68	9.68	9.63	9.57	9.57
Proposed RF/DALI ballast																	
Energy (kWh)		5.83	5.82	5.78	5.72	5.71	5.65	5.60	5.60	5.54	5.54	5.49	5.49	5.49	5.45	5.41	5.41
Cost (€)		0.93	0.93	0.92	0.92	0.91	0.90	0.90	0.90	0.89	0.89	0.88	0.88	0.88	0.87	0.87	0.87
Non regulated ballast																	
Energy (kWh)		9.18	9.16	9.10	9.03	9.01	8.94	8.86	8.86	8.79	8.79	8.71	8.71	8.71	8.67	8.61	8.61
Cost (€)		1.47	1.47	1.46	1.44	1.44	1.43	1.42	1.42	1.41	1.41	1.39	1.39	1.39	1.39	1.38	1.38
Savings	(%)	36.47	36.50	36.56	36.64	36.69	36.74	36.84	36.85	36.93	36.96	37.04	37.06	37.06	37.15	37.14	37.14
Clock regulated 2-level ballast																	
Energy (kWh)		6.41	6.40	6.36	6.32	6.31	6.26	6.22	6.22	6.17	6.17	6.13	6.13	6.13	6.10	6.07	6.07
Cost (€)		1.03	1.02	1.02	1.01	1.01	1.00	1.00	1.00	0.99	0.99	0.98	0.98	0.98	0.98	0.97	0.97
Savings	(%)	8.99	9.05	9.22	9.45	9.53	9.72	9.96	9.99	10.20	10.25	10.47	10.50	10.50	10.69	10.78	10.78

**Table 3:** Estimated consumption (energy and cost) for 1-year operation.

Regulation Mode	ATR	DBL	UCODALI
Energy (kWh)	3879.315	2809.642	2663.704
Cost (€)	682.76	449.54	426.19

**Table 4:** Saving rates (energy and cost) for 1-year operation.

UCODALI Savings	*vs.* ATR		*vs.* DBL	
Energy (kWh)	1215.611	31.34%	145.938	5.19%
Cost (€)	256.57	37.58%	23.35	5.19%
